# National outcomes of expedited discharge following esophagectomy for malignancy

**DOI:** 10.1371/journal.pone.0297470

**Published:** 2024-02-23

**Authors:** Shayan Ebrahimian, Nikhil Chervu, Joseph Hadaya, Nam Yong Cho, Elsa Kronen, Sara Sakowitz, Arjun Verma, Syed Shahyan Bakhtiyar, Yas Sanaiha, Peyman Benharash

**Affiliations:** 1 Cardiovascular Outcomes Research Laboratories, Division of Cardiothoracic Surgery, David Geffen School of Medicine at UCLA, Los Angeles, CA, United States of America; 2 Department of Surgery, Division of Cardiac Surgery, David Geffen School of Medicine at UCLA, Los Angeles, CA, United States of America; University of Florida, UNITED STATES

## Abstract

**Background:**

Expedited discharge following esophagectomy is controversial due to concerns for higher readmissions and financial burden. The present study aimed to evaluate the association of expedited discharge with hospitalization costs and unplanned readmissions following esophagectomy for malignant lesions.

**Methods:**

Adults undergoing elective esophagectomy for cancer were identified in the 2014–2019 Nationwide Readmissions Database. Patients discharged by postoperative day 7 were considered *Expedited* and others as *Routine*. Patients who did not survive to discharge or had major perioperative complications were excluded. Multivariable regression models were constructed to assess association of expedited discharge with index hospitalization costs as well as 30- and 90-day non-elective readmissions.

**Results:**

Of 9,886 patients who met study criteria, 34.6% comprised the *Expedited* cohort. After adjustment, female sex (adjusted odds ratio [AOR] 0.71, p = 0.001) and increasing Elixhauser Comorbidity Index (AOR 0.88/point, p<0.001) were associated with lower odds of expedited discharge, while laparoscopic (AOR 1.63, p<0.001, Ref: open) and robotic (AOR 1.67, p = 0.003, Ref: open) approach were linked to greater likelihood. Patients at centers in the highest-tertile of minimally invasive esophagectomy volume had increased odds of expedited discharge (AOR 1.52, p = 0.025, Ref: lowest-tertile). On multivariable analysis, expedited discharge was independently associated with an $8,300 reduction in hospitalization costs. Notably, expedited discharge was associated with similar odds of 30-day (AOR 1.10, p = 0.40) and 90-day (AOR 0.90, p = 0.70) unplanned readmissions.

**Conclusion:**

Expedited discharge after esophagectomy was associated with decreased costs and unaltered readmissions. Prospective studies are necessary to robustly evaluate whether expedited discharge is appropriate for select patients undergoing esophagectomy.

## Introduction

Despite incremental refinements in patient selection, surgical technique, and perioperative management, esophagectomy remains associated with substantial morbidity and resource utilization [1]. Our group has previously demonstrated a positive volume-outcome relationship in a myriad of complex surgical operations [2–6]. Technical expertise aside, enhanced quality of care at experienced institutions may be attributable to the presence of standardized care pathways that reduce expenditures while optimizing clinical outcomes.

Expedited discharge represents one such perioperative care strategy that is commonly implemented at high volume centers and has garnered significant interest in recent years. Several studies have examined expedited discharge following cardiac and thoracic operations, reporting decreased expenditures and complications without an increase in readmission rates [7–9]. With a recent study noting an average hospitalization duration of 14 days following esophagectomy, postoperative length of stay appears to be a suitable target for improving value in the surgical management of esophageal cancer [10]. Several groups have reported encouraging outcomes with implementation of Enhanced Recovery After Surgery (ERAS) protocols following esophagectomy while targeting discharge ≤ 7 days [11–14]. In contrast, others have noted a paradoxical increase in healthcare costs, readmissions, and adverse events with this approach [10, 15, 16].

In the absence of national benchmarks regarding the feasibility and cost-effectiveness for expedited discharge, a large-scale study of this strategy in esophagectomy is warranted. Therefore, we used a national cohort of uncomplicated patients with esophageal cancer to examine the association of expedited discharge with short-term clinical and financial outcomes following esophagectomy. We hypothesized expedited discharge to be associated with reduced resource use and unaltered risk of 90-day, unplanned readmission.

## Materials and methods

### Data source and study population

The present study was a retrospective cohort study of the 2014–2019 Nationwide Readmission Database (NRD). The NRD is the largest readmissions database in the United States (US) and is maintained as part of the Health Care Cost and Utilization Project (HCUP) [17]. Using validated survey weighting methodology, the NRD provides accurate estimates for approximately 57% of all US hospitalizations [17]. Moreover, the NRD uses unique patient and hospital identifiers to track admissions across participating centers within each calendar year. This study was deemed exempt from full review from the Institutional Review Board (IRB) at the University of California, Los Angeles (IRB#17–001112). Patient consent (verbal or written) was also waived due to the de-identified nature of the database.

All elective adult (≥18 years) hospitalizations entailing esophagectomy for malignancy were identified using the International Classification of Diseases, Ninth and Tenth revisions codes (ICD-9/10) [18]. Patients who did not survive to discharge, who developed major perioperative complications (cardiac, respiratory, infectious, gastrointestinal, neurological, renal, and thromboembolic complications) or who were discharged >15 days following esophagectomy (90^th^ percentile for postoperative length of stay) were excluded from analysis. To ensure adequate 90-day follow up, we further excluded discharges in the months of October, November, and December, for each year. Those with missing key records were also excluded (n = 603, 6% of cohort). Patients were stratified as *Expedited* if discharged on or before postoperative day 7, in agreement with prior work and corresponding to the lowest quartile of postoperative length of stay on exploratory analysis [11–14]. The remaining patients were classified as *Routine*.

#### Variable definitions and outcomes

Patient and hospital characteristics such as age, income level, and hospital region were defined according to the HCUP data dictionary [17]. The Elixhauser Comorbidity Index, a validated composite score of 30 conditions, was used to quantify the burden of chronic illness [19]. Other comorbidities and complications were ascertained using previously reported ICD-9/10 codes [20]. Operative approach was similarly defined and categorized into open, laparoscopic and robotic-assisted. Hospitals were designated as low-, medium- and high-volume institutions based on the 33^rd^ and 66^th^ percentiles of total annual esophagectomy caseload. Hospitals were further categorized into tertiles based on the annual minimally invasive (MIS) and open esophagectomy case volume. Hospitalization costs were calculated by applying center-specific cost-to-charge ratios to total hospitalization charges and adjusted for inflation using the 2019 Personal Health Care Index [17]. The primary outcome of interest was index hospitalization cost, while secondary outcomes included 30-day and 90-day unplanned readmissions.

#### Statistical analysis

Categorical variables are reported as proportions and were compared using Pearson Chi-square test. Continuous variables with normal distribution are summarized as means with standard deviation while skewed continuous variables are reported as medians with interquartile range (IQR). The Adjusted Wald and Mann-Whitney U tests were utilized to compare means and medians, respectively. Cuzick’s non-parametric test of trend (nptrend) was used to assess changes in operative approach over the study period [21]. Royston-Parmar parametric regression was used to visualize freedom from unplanned readmission within 90-days of index discharge [22]. Multivariable regression models were developed to evaluate the independent association of covariates with outcomes of interest. Elastic Net regularization, which employs a regressive least squares methodology, was utilized for variable selection to reduce bias and improve the out-of-sample generalizability [23]. Following retention of clinically relevant variables, final models were selected using Akaike information criteria and receiver operating characteristics. Regression outputs are reported as adjusted odds ratios (AOR) or beta coefficients (β) with 95% confidence intervals (95% CI). Statistical significance was set as α = 0.05. All statistical analyses were performed using Stata 16.1 (StataCorp, College Station, TX).

## Results

### Demographics and unadjusted outcomes

Of an estimated 9,886 hospitalizations meeting study criteria, 34.6% comprised the *Expedited* cohort. Patients were hospitalized for a median of 8 days (IQR 7–10) following surgery. The median postoperative length of stay for the *Expedited and Routine* cohorts were 7 and 9 days respectively. Patients in the *Expedited* cohort were less frequently female (15.3 vs 19.1%, p = 0.001), more commonly privately insured (46.0 vs. 42.7%, p = 0.010), and had a lower estimated burden of comorbidities as measured by Elixhauser Index (3 [IQR 2–4] vs 4 [IQR 3–5], p<0.001). In addition, *Expedited* patients more commonly underwent laparoscopic (25.9 vs 17.4%, p<0.001) and robotic (17.9 vs. 11.1%, p<0.001) esophagectomy compared to *Routine*.

*Expedited* patients more frequently underwent esophagectomy at highest volume tertile hospitals and Metropolitan teaching centers (Table 1). Additional patient characteristics for the *Expedited* and *Routine* cohorts are shown in Table 1.

**Table 1:** Bivariate comparison of baseline characteristics of patients undergoing esophagectomy for malignancy stratified by expedited discharge. *SD*: *Standard Deviation; IQR*: *interquartile range*.

	Routine (n = 6,460)	Expedited (n = 3,426)	p-Value
Age (year, mean ± SD)	63.8 ± 9.7	63.2 ± 9.9	0.06
Female (%)	19.1	15.3	0.001
*Income Quartile (%*, *Percentile)*			0.2
76^th^-100^th^	24.4	26.1	
51^st^-75^th^	27.1	29.2	
26^th^-50^th^	28.1	26.6	
0^th^ -25^th^	20.4	18.1	
*Insurance Type (%)*			0.01
Private	42.7	46.0	
Medicare	47.6	44.6	
Medicaid	6.2	6.2	
Other payer	3.5	3.2	
*Comorbidities (%)*			
Elixhauser Comorbidity Index (median, IQR)	4 (3–5)	3 (2–4)	<0.001
Congestive heart failure	4.2	4.3	0.9
Arrhythmia	34.5	27.3	<0.001
Peripheral vascular disorder	4.3	3.9	0.6
Hypertension	55.2	52.0	0.06
Chronic lung disease	17.7	15.0	0.02
Hypothyroidism	7.7	8.2	0.7
Liver disease	4.4	4.8	0.5
Coagulopathy	6.4	4.9	0.05
Diabetes	21.1	19.1	0.1
*Cancer Location (%)*			0.08
Upper third of esophagus	2.2	1.8	
Middle third of esophagus	9.1	8.3	
Lower third of esophagus	65.4	61.9	
Overlapping/unspecified	23.3	28.0	
*Operation type (%)*			<0.001
Open	71.5	56.2	
Laparoscopic	17.4	25.9	
Robotic	11.1	17.9	
*Hospital Location/Teaching Status (%)*			0.03
Non-metropolitan	0.8	0.3	
Metropolitan non-teaching	4.9	3.7	
Metropolitan teaching	94.2	96.0	
*Hospital Total Operative Volume Tertiles (%)*			0.02
Lowest	26.8	20.8	
Middle	31.1	37.4	
Highest	42.1	41.8	

**Fig 1:**
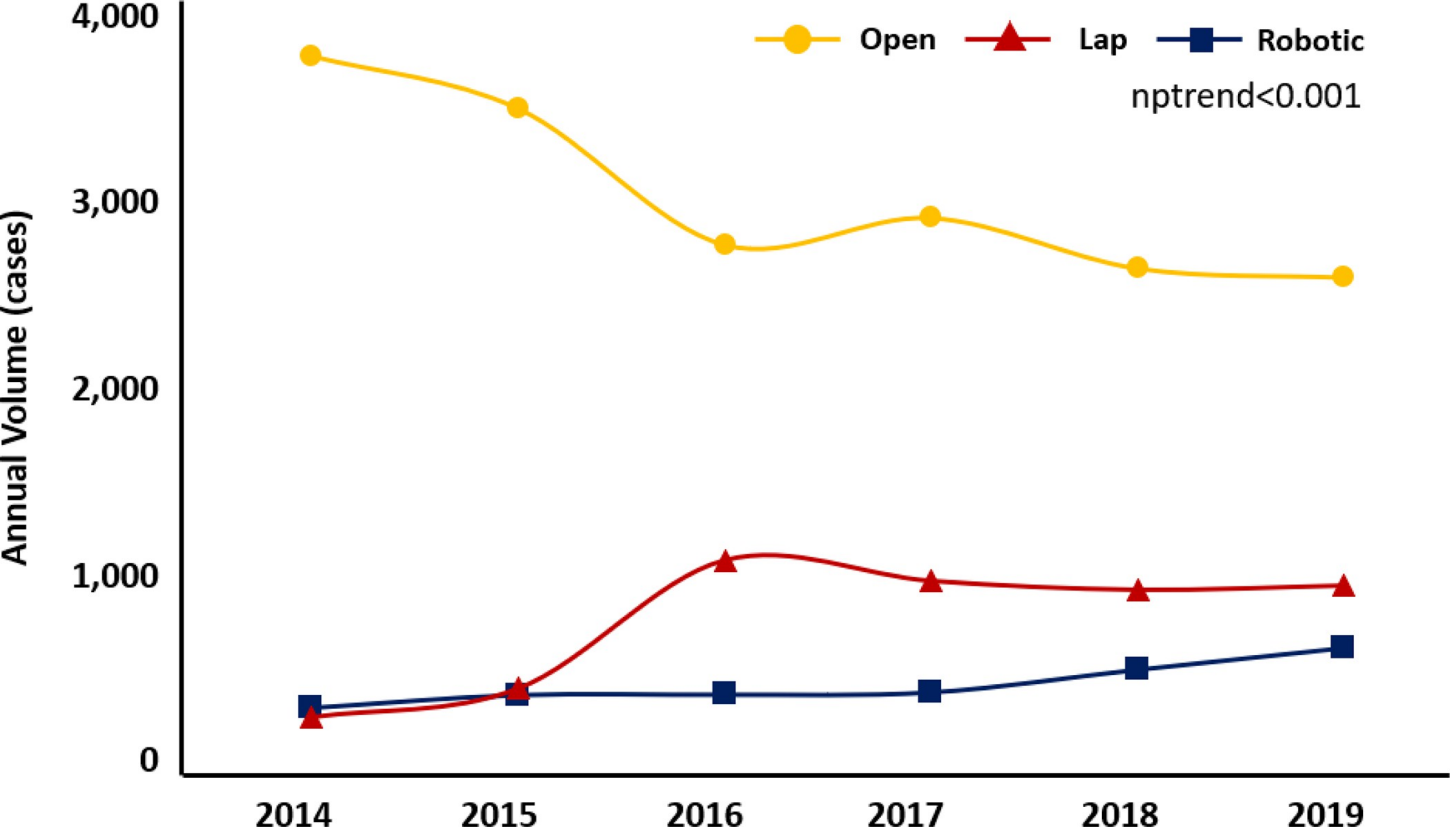
Temporal trends in annual volume of esophagectomy for malignancy stratified by operative approach. Annual *volume of laparoscopic (red) and robotic (blue) approaches increased*, *while the overall volume of esophagectomy remained similar over the study period*. *The non-parametric test of trend (nptrend), *P<0*.*0001*.

While the overall volume of esophagectomy remained similar over the study period, the annual volume of laparoscopic and robotic approaches prevalence of laparoscopic and robotic approach increased (nptrend <0.001, Fig 1). The Median postoperative length of stay stratified by operative approach (Open: 9 days, Lap: 8 days, Robotic: 8 days) also remained unchanged over the study period. Compared to *Routine*, patients in the *Expedited* cohort accrued significantly lower unadjusted index hospitalization costs ($32,200 [IQR 24,900 - $41,000] vs $38,800 [29,800 - $50,600]) but had similar unadjusted rates of 30-day (13.0 vs 12.8%, p = 0.8**0**) and 90-day readmissions (17.8 vs 19.4%, p = 0.20, Table 2).

**Table 2:** Crude and risk-adjusted outcomes after esophagectomy stratified by expedited discharge. AOR: Adjusted Odds Ratio. Bivariate comparisons are reported as percentages or medians with interquartile range [IQR]. Risk-adjusted estimates are reported as Beta coefficients (β) and adjusted odds ratio (AOR) for continuous and binary and variables, respectively.

	Routine	Expedited	P-Value	AOR/ß [95% CI]	p-Value
	(n = 6,460)	(n = 3,426)			
***Resource Utilization (Median [IQR]*, *AOR/ß*** *)*					
Length of stay (days)	9 [8 – 11]	7 [6 – 7]	<0.001	–3.3 [–3.5 - –3.1]	<0.001
Hospitalization costs ($1,000s)	38.8 [29.8–50.6]	32.2 [24.9–41.0]	<0.001	–8.3 [–9.6 - –7.1]	<0.001
30-day, Non-elective readmissions	12.8	13.0	0.80	1.1 [0.8–1.4]	0.40
90-day, Non-elective readmissions	19.4	17.8	0.20	0.9 [0.8–1.2]	0.70

### Association of patient factors and hospital volume with expedited discharge

**Fig 2:**
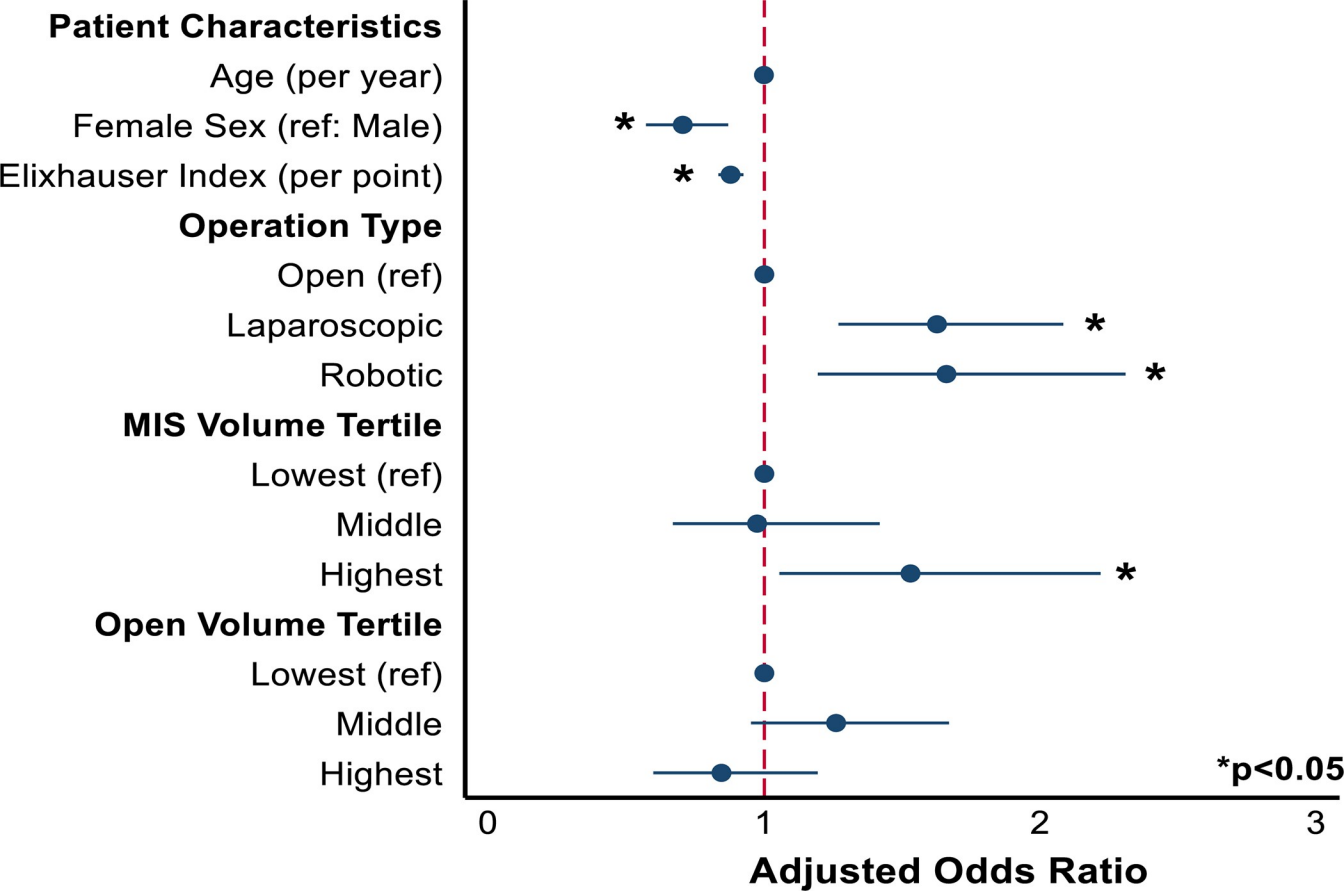
Coefficient plot of patient, operative, and hospital characteristics associated with expedited discharge following esophagectomy for malignancy. MIS Volume: Minimally invasive surgery hospital volume; Open Volume: Open surgery hospital volume.

A multivariable logistic regression model (C-statistic: 0.67) was developed to examine factors associated with expedited discharge (Fig 2). Following adjustment for intergroup differences, female sex (AOR 0.71, 95% CI 0.57, 0.87) and increasing Elixhauser Comorbidity Index (AOR 0.88/point, 95% CI 0.83, 0.92) were associated with lower odds of expedited discharge (Fig 1). Relative to open, laparoscopic (AOR 1.63, 95% CI 1.27, 2.09) and robotic (AOR 1.67, 95% CI 1.20, 2.32) approaches were associated with greater likelihood of expedited discharge. Of note, patients at centers in the highest volume tertile for MIS esophagectomy had increased odds of expedited discharge (AOR 1.52, 95% CI 1.05, 2.21, Ref: lowest MIS tertile). Open volume caseload did not alter the odds of expedited discharge (highest open tertile: AOR 0.85, 95% CI 0.60, 1.19; middle open tertile: AOR 1.26 95% CI 0.95, 1.67; Ref: lowest open tertile).

### Outcomes associated with expedited discharge

**Fig 3:**
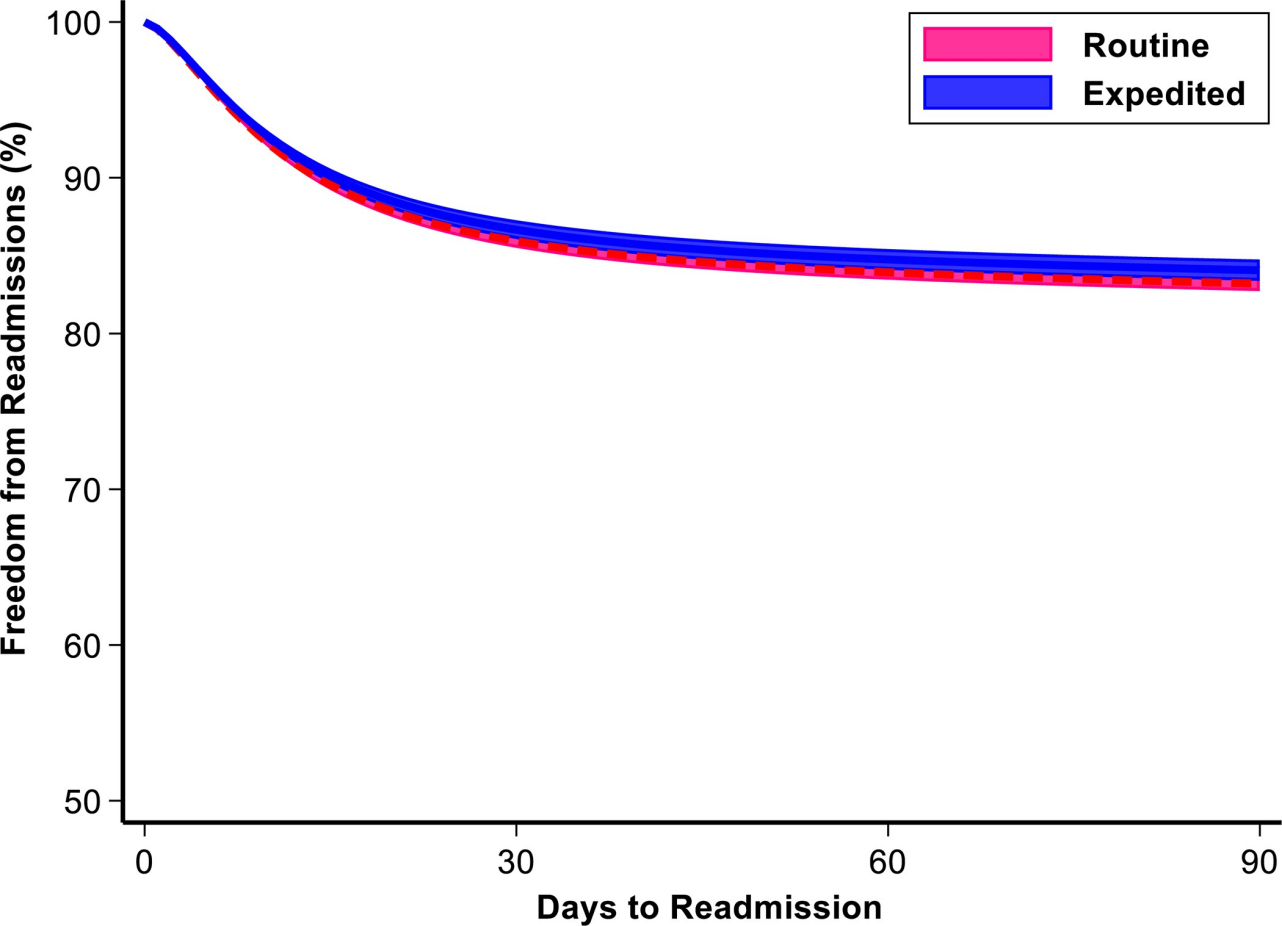
Adjusted odds of freedom from readmission across 90 days post-discharge using royston parmar modeling. Freedom from readmission is defined as an inverse measure of the odds of unplanned readmission. Red dash line represents the risk of the Routine cohort, and the blue solid line denotes the risk for Expedited cohort.

After multivariable adjustment, expedited discharge remained independently associated with lower index hospitalization costs (ß –8,300, 95% CI–$9,600,–$7,100) but similar odds of 30-day (AOR 1.10, 95% CI 0.80, 1.40) and 90-day readmission (AOR 0.90, 95% CI 0.80, 1.20), with routine discharge as reference (Table 2 and Fig 3). Based on these results, if all uncomplicated patients were to undergo expedited discharge following esophagectomy, the total cost saving would be approximately $53,600,000 across the 6-year study period.

## Discussion

Despite advances in surgical technique and postoperative care, esophagectomy for malignancy remains associated with substantial risk of major morbidity, prolonged hospitalization, and costs. In the present national analysis, we examined factors associated with expedited discharge (≤7 days postoperative duration of stay) following esophagectomy, as well as the impact of expedited discharge on costs and rates of readmission. We found several factors including minimally invasive approaches and increased institutional MIS volume to be independently associated with increased odds of expedited discharge. Of note, expedited discharge was linked to a substantial reduction in episodic costs, amounting to more than $50 million in overall cost savings over the 6-year study period. We did not find any significant association between expedited discharge and the risk of unplanned rehospitalization. Several of these findings warrant further discussion.

Enhanced recovery after surgery programs aim to standardize postoperative operation-specific care pathways and reduce risk of complications through measures such as timely removal of drains and appropriate perioperative antibiotic prophylaxis [11–14, 24]. Such programs have been readily implemented in various surgical disciplines ranging from colorectal to cardiac surgery [25, 26]. Limited studies have examined expedited discharge or enhanced recovery after surgery programs following esophagectomy, partly due to the limited available sample size and lack of national benchmarks for length of stay. In the present national analysis of uncomplicated esophagectomy hospitalizations, we noted a median postoperative length of stay of 8 days, with only 34.6% of patients discharged on or before postoperative day 7. The low utilization of expedited discharge in this national cohort warrants further study as several groups have reported success with accelerated pathways [11–14]. Perioperative measures such as multimodal pain management, restricted fluid administration, and early feeding are most common among enhanced recovery programs [11–14]. In the case of esophagectomy, a substantial degree of surgeon and hospital level variation is likely present. Various protocols for postoperative feeding and radiographic assessment for anastomotic leak prior to initiating enteral nutrition are utilized across the country without consensus. For example, Tomaszek et al. found that avoidance of a routine contrast swallow study, maintenance of feeding jejunostomy tube, and delaying oral intake until postoperative week 4 was associated with a reduction in length of stay by 2 days, without an increase in post-discharge complications [27]. Similarly, in a single-institution study, use of a preoperative nutrition regimen prior to esophagectomy was associated with reduced complications and shorter length of stay [28]. Although we were unable to evaluate factors such as jejunostomy placement, preoperative nutrition, or the presence of an ERAS program at specific hospitals, our results suggest that expedited discharge following esophagectomy is achievable in select patients and not associated with excess morbidity or readmissions. Further work to develop comprehensive postoperative pathways and increase their adoption may facilitate expedited discharge in appropriately selected candidates.

Patient risk profile as well as institutional practice patterns are known to influence morbidity following surgery as well as expected postoperative length of stay [29, 30]. In our analysis, we found several operative and hospital factors to be associated with increased odds of expedited discharge. Minimally invasive approaches such as robotic and laparoscopic surgery were associated with greater odds of expedited discharge. This finding may be related to early mobility and reduced postoperative pain, which may be facilitated by minimally invasive surgery. Notably, greater institutional MIS operative volume was independently associated with greater odds of expedited discharge. Consistent with our results, prior studies have demonstrated institutional volume and surgeon experience to be associated with improved outcomes, reduced operative times, and reduced costs in both cardiac and thoracic operations [29, 31]. These effects are likely due to standardized institutional care pathways that reduce variability in care and more efficiently provide cost-effective care at the physician, nursing, and ancillary staff level. As such, referral of esophagectomy cases to high-volume centers may reduce complications which are major drivers for prolonged hospitalization. Continued development of institutional, societal or national guidelines, as well as work to facilitate implementation of care pathways may improve patient outcomes and reduce costs of care at the large scale.

In our analysis, we found that expedited discharge was linked to a $8,300 reduction in index costs without an increase in the odds of 30-day or 90-day readmission. This degree of cost savings may be related to both overall more efficient care, as well as a reduction in the daily costs of inpatient hospitalization. These cost savings are consistent with prior work examining isolated coronary artery bypass grafting [8] and pulmonary resection [7], suggesting that expedited discharge can be considered a goal for appropriately selected patients. Unplanned readmission, development of complications as an outpatient, and reduced patient satisfaction have been cited as concerns regarding expedited discharge of patients undergoing complex surgery [10, 15, 16]. Interestingly, Sundaram and colleagues reported decreased odds of readmission among patients undergoing esophagectomy for esophageal cancer who had a longer index length of stay compared to those with a short index length of stay using the National Quality Improvement Program (NSQIP) database [10]. However, this analysis was limited to 30-days following surgery rather than discharge, due to the structure of the NSQIP database [10]. In the present study, the use of the NRD allowed for independent examination of readmissions following discharge at the national level rather than select NSQIP hospitals which are often high-performing and do not represent care nationally [17, 32]. Future prospective studies are necessary to better evaluate the impact of expedited discharge pathways among patients undergoing esophagectomy.

The present work has several important limitations including its retrospective nature and use of an administrative database. Causation cannot be inferred due to the observational nature of the study. We could not capture important factors such as operative time, type of resection, blood loss, interval to oral feeding, stage of cancer, outpatient healthcare utilization or patient satisfaction. Moreover, we were unable to delineate specific reasons for longer length of stay that are likely related to individual patient factors such as operative complexity, morbidities not captured in the dataset, as well as social determinants of health. Despite inherent limitations of the study design and data source, we used rigorous statistical methods and a national sample to reduce the risk of bias and evaluate expedited discharge on a national basis in an all-payer database.

## Conclusion

Expedited discharge following esophagectomy is uncommon, reduces index hospitalization costs, and does not increase rehospitalization within three months of discharge. Although prospective studies are ultimately needed, our findings suggest the relative safety of expedited discharge following esophagectomy and may provide support for the use of minimally invasive approaches.
